# No Incongruity in Respiratory Syncytial Virus M2-1 Protein Remaining Bound to Viral mRNAs during Their Entire Life Time

**DOI:** 10.1128/mBio.00187-19

**Published:** 2019-05-07

**Authors:** Marie-Anne Rameix-Welti

**Affiliations:** a Université Versailles Saint Quentin en Yvelines, Montigny-le-Bretonneux, France; Weill Cornell Medicine; Catholic University of America

**Keywords:** M2-1, respiratory syncytial virus, transcription

## LETTER

Selvaraj et al. recently published the structure of the M2-1 protein of respiratory syncytial virus (RSV) bound to its interaction domain on the P protein (1). These results constitute an important step toward the understanding of RSV transcription process and, on a broader level, of RSV RNA-dependent RNA polymerase regulation and functioning. Interestingly, their results suggest that M2-1 could play a posttranscriptional role in viral mRNA metabolism which is consistent with our previous description of concentration of newly synthetized viral mRNAs together with M2-1 in inclusion body-associated granules (IBAGs) (2). In this model, M2-1 would remain bound with viral mRNAs in the cytosol to regulate mRNA translation and/or stability. However, they point out the small amount of M2-1 as a critical incongruity of this model. M2-1 is indeed the ninth (next to the last) ORF in the RSV genome and is expected to be less transcribed due the transcriptional gradient observed in *Mononegavirales*. Thus, they suggest that there would not be enough M2-1 tetramers to bind to all viral mRNAs at one point in an infected cell. I respectfully disagree with this statement. Using a high-resolution transcriptomic approach, Aljabr et al. indeed show that at 24 h postinfection, the M2 mRNAs accounted for around 4% of the viral mRNAs (3). However, the translation rate of RSV mRNAs is unknown. In mammalian cells, the median translation rate of a messenger would be 140 proteins per hour, and the ratio of average protein to mRNA has been found to be around 2,800 (4). If we take as a rough assumption that viral mRNAs are translated as efficiently as average cellular mRNAs, one M2 mRNA would generate 2,800 M2-1 proteins. This would result in 28 tetramers of M2-1 per viral mRNA. Thus, there would be more than enough M2-1 tetramers in the cell to bind with all viral mRNAs. In mammalian cells, proteins are on average five times more stable than mRNAs, suggesting that M2-1 may also be recycled, as raised by Selvaraj et al. (1). Applying the 140 proteins per hour average translation rate to viral mRNA would also solve the apparent contradiction of the first transcription round. Indeed, assuming that the M2-1 remains associated with newly synthetized viral mRNA, about 25 M2-1 tetramers would theoretically be enough to allow synthesis of M2 mRNA. The relative level of M2-1 no longer being an obstacle, a model in which M2-1 would stay associated with viral mRNA during their whole life is the best fit with published results. The next challenge is now to determine the role of such association in regulating viral RNAs translation and stability.
